# Supplementary material for: DNAJA3 Interacts with PEDV S1 Protein and Inhibits Virus Replication by Affecting Virus Adsorption to Host Cells
Source: Viruses. 2022 Oct 31;14(11):2413. doi: 10.3390/v14112413 (PMC9696540; doi:10.3390/v14112413)
Supplement: Supplementary file 1 [file viruses-14-02413-s001.zip › viruses-1979131-supplementary.pdf]

Supplementary Material

1 Supplementary Figure S1

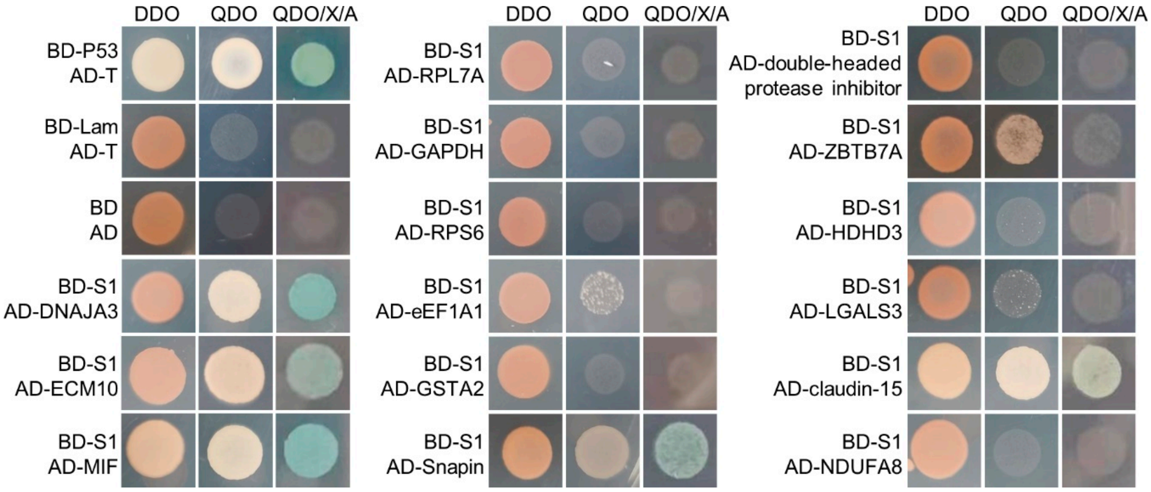

Figure S1. Host proteins that interact with PEDV S1 protein as verified by yeast two-hybrid assay. Yeast strain Y2HGold was co-transformed with bait plasmid pGBKT7-S1 (BD-S1) and prey plasmid that screened from the pGADT7-based porcine cDNA library. The blue bacterial lawn growing on the QDO/X/A plates represents positive interactions.
